# Role of gp91*phox* Homolog Nox1 in Induction of Premalignant Spindle Phenotypes of HPV 16 E6/E7—Immortalized Human Keratinocytes

**DOI:** 10.1100/tsw.2010.131

**Published:** 2010-07-20

**Authors:** Walee Chamulitrat

**Affiliations:** Internal Medicine IV, Department of Gastroenterology and Infectious Disease, University Heidelberg Hospital, Germany

**Keywords:** NADPH oxidase, reactive oxygen species, epithelial cytokeratin, vimentin, tumor progression, invasion, MAPK activation, inducible nitric oxide synthase

## Abstract

The NADPH oxidase (Nox) family of superoxide- and hydrogen peroxide—producing proteins has been recognized as important for signal transduction that regulates receptor-mediated functions, including cytoskeleton remodeling, cell proliferation, migration, differentiation, and cell death. Nox1 was the first of the Nox catalytic subunits to be cloned and shown to induce tumorigenic conversion of mouse fibroblasts. While Nox1 has been shown to be expressed in human colon and prostate cancers, however, limited studies have demonstrated the involvement of Nox1 in an early step of cell transformation. The aim of this review is to provide an overview on the role of Nox1 in cancer, as well as the contribution of our studies to demonstrate the involvement of Nox1 on neoplastic progression of human keratinocytes beyond the immortalization step. The generation of spindle phenotypes concomitant with anchorage-independent growth and invasiveness will be highlighted and discussed in relation to the possible role of Nox1 in epithelial-mesenchymal transition. Understanding these mechanisms may provide insight into Nox1 and redox signaling components as potential therapeutic targets to inhibit tumor progression.

## INTRODUCTION

Reactive oxygen species (ROS) are conventionally thought to be cytotoxic and mutagenic, and in high levels they induce cell death, apoptosis, and senescence. In contrast, ROS at low levels function as signaling molecules to mediate cell growth, migration, differentiation, and gene expression. Elevated levels of ROS, namely, superoxide and hydrogen peroxide, are found in human tumor cells[1] and malignantly progressed keratinocytes[2]. Mitochondrial ROS, which are produced by the respiratory chain, have been thought to act as prosurvival and prodeath signals exerting a decisive control over several biochemical cascades, leading to cell death and the intrinsic pathway of apoptosis[3]. ROS could serve as an endogenous source of DNA-damaging agents to promote genetic instability. In addition, several mitochondrial DNA mutations were reported in tumors and the construction of cybrids recently demonstrated their role in the control of tumor progression[4]. Besides mitochondria, enzymes involving redox regulation have also been implicated in cancer, including polyamine oxidases[5], lipoxygenases[6,7], and NADPH oxidase gp91*phox* (Nox) homologs[8]. Nox proteins are membrane-associated, multiunit enzymes that catalyze the reduction of oxygen using NADPH as an electron donor. Nox proteins produce a superoxide radical by a single electron reduction. NADPH oxidase historically is known to be the source of ROS during phagocytosis. In the past 15 years, Nox family members (Nox 1–5 and Duox 1/2) present in a variety of tissues have been identified as important mediators of normal physiologic functions that include innate immunity, signal transduction, and biochemical reactions; for example, to produce thyroid hormone[9]. With the high biochemical reactivity of ROS, Nox enzymes have been implicated in a wide variety of pathologies and diseases, particularly those associated with inflammation, aging, and progressive degenerative changes in cells and organ systems. Nox-derived ROS are involved in chronic diseases that tend to appear later in life, including atherosclerosis, hypertension, diabetic nephropathy, lung fibrosis, Alzheimer's disease, and cancer[10]. Nox enzymes may thus be the targets of drugs for treatment of these chronic diseases[11]. Compared with other Nox isoforms, the most intensively studied, Nox1, has been shown to be highly expressed in human colon and prostate cancers[8,12,13]. This review summarizes our findings to implicate Nox1 in preneoplastic progression by an induction of epithelial-mesenchymal transition (EMT) of immortalized human keratinocytes. Signaling pathways induced by Nox1 in alterations of cytoskeletal remodeling will be discussed.

## NOX1 AS A SOURCE OF ROS IN CANCER

During 1999–2000, three papers reported the first sequence of gp91*phox* homolog cDNA, all cloned from normal human colon[8,14,15]. This gene was formally called Mox1 (mitogenic oxidase1) and later renamed to Nox1. All Nox isoforms are expressed on the mRNA level in fetal tissues more abundantly than in adult tissues, and Nox 1, 2, 4, and 5 mRNAs are expressed in a variety of human cancer cell lines, indicating an association with cancer development[16]. Nox1, a homolog of gp91*phox*, is described as highly expressed in human colon, but at lower levels in vascular smooth muscle, endothelial cells, uterus, placenta, retinal pericytes, neurons, astrocytes, and microglia. Nox1 is capable of mediating tumorigenic conversion when overexpressed in rodent NIH3T3 fibroblasts[8]. In these cells, overexpression of Nox1 increases tumorigenic potentials and catalase overexpression reverses tumor promotion[17]. This indicates that hydrogen peroxide generated secondarily by the enzyme serves as the transformation signal.

For the case of human epithelial cancer cells, overexpression of Nox1 increases tumorigenic potentials of the DU-145 human prostate cancer cell line[18], which is supported by Nox1 presence in human prostate cancer tissues[13]. Prostate tumors are significantly more likely to have Nox1 staining than benign prostate tissues[19]. Nox1 mRNA is present in both primary and transformed human colonic epithelial cells[20], while Nox1 mRNA and protein are overexpressed in human colonic tumors compared with paired normal tissue as early as the adenoma stage with no correlation of expression level with tumor stage[12]. Other data also indicated that Nox1 may induce differentiation of colonic cancer cells HT-29[21], and thus is highly expressed in well-differentiated colon cancer[22]. Nox1 may play a distinct role during cell differentiation of normal and tumor tissues. Nox1 not only plays a role in colonic carcinogenesis, but also in innate immune response of the gastrointestinal tract[23]. Accordingly, Nox1 detected in colon epithelial crypts and on the luminal surface is implicated as playing a role in host defense function[24]. In addition to the known epithelial localization, lymphocytes are a novel site of Nox1 expression, where it may potentially be involved in the pathogenesis of inflammatory bowel diseases[24]. It is known that activation of NADPH oxidases in intestinal lymphocytes generate ROS[25]. In this light, additional insights have been raised that Nox1-generated ROS in lymphocytes may induce colonic inflammation, which predisposes the colon for neoplastic progression. In addition to colon and prostate cancers, Nox1 also stimulates proliferation of breast and ovarian cancer cells by mediating a cross-talk with mitochondria[26]. In mammalian epithelial cells, chronic oxidative stress induced by Nox1 appears to saturate cellular DNA repair capacity and cause significant genomic instability, which may lead to neoplastic progression[27].

## Molecular Structure and Regulation of Nox1

The Nox1 gene contains 564 amino acids[8]. Two splicing forms of Nox1 were described as Nox-1S (shown to mediate $H^+$ current) and Nox-1L-tv (Nox1 long-truncated variant)[14]. However, Nox-1S was later identified as a cloning artifact during RNA amplification[28]. Nox-1L-tv has 49 amino acids (residues 431 to 488 containing one of the four NADP-binding sites) in exon 11 missing from the *bona fide* Nox1. This missing NADP-binding site containing $^{435}$FYWICRE$^{441}$ is highly conserved and shared by other Nox enzymes[14]. Nox-1L-tv is detected in human colon, uterus, prostate, and Caco-2 cells. While the function of Nox-1L-tv (or Nox1-tv) is not known, it was reported that Nox1-tv is not a functional enzyme as it did not support ROS generation in a presence of Nox1-supportive cofactors (NoxO1 and NoxA1)[29,30,31]. Thus, the exon 11–deleted Nox1-tv transcript does not encode functional oxidase despite its abundance in various tissues.

Nox1 contains six transmembrane domains (Fig. 1). Oxidase activity occurs when NADPH binds to Nox1 on the cytosolic side, where it transfers its electrons to FAD and hemes, and finally to oxygen on the outer membrane surface to form a superoxide radical. Nox1 associates with the membrane subunit p22*phox*, which is necessary for Nox1 activation[32]. In a similar manner as gp91*phox* (Nox2), Nox1 can interact with p47*phox* and p67*phox*, but is most highly activated by the respective homologs NoxO1 and NoxA1[29,30,31]. In contrast to p47*phox*, NoxO1 is constitutively associated with Nox1. Similar to Nox2 activation, NoxA1[33] and GTP-activated Rac1 membrane translocation[34,35] are required for Nox1 activation and initiation of superoxide generation.

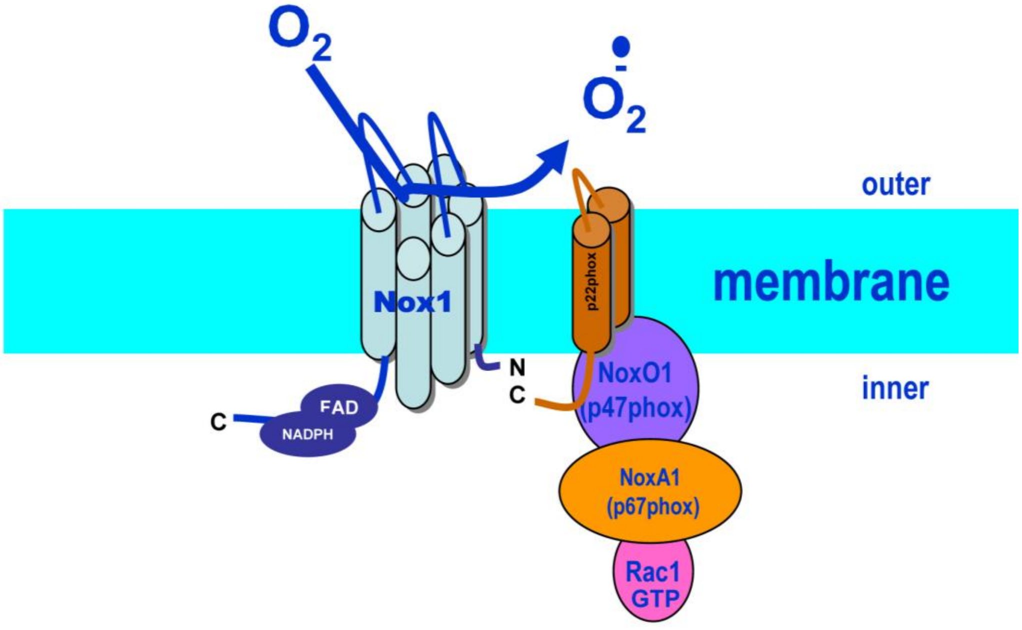

**FIGURE 1**. Nox1 and its regulatory subunits NoxO1, NoxA1, and Rac1, as well as the assembly factor p22*phox*.

## Subcellular Localization of Nox1

Depending on cell type, various Nox1 localizations have been reported. In the plasma membrane fraction from Caco-2 human colon carcinoma cells, active Nox1 endogenously colocalizes with its assembly factor p22*phox* and regulatory components NoxO1, NoxA1, and Rac1[33]. In intermediate transformed keratinocytes, we showed that Nox1 was found to have a nuclear localization with some cytoplasmic distribution[36]. In vascular smooth muscle cells, distribution of Nox1 on plasma membrane, specifically caveolae, as well as in the nucleus has been reported[37]. By forced expression, Nox1 is found to be localized on the plasma membrane[38,39]. It appears that the very N-terminal part of Nox1 determines subcellular localization, whereas the cytosolic tail regulates activity[39]. It is conceivable that Nox1 localization on the specialized microdomain of plasma membrane caveolae and lipid rafts supports activation of the epidermal growth factor receptor that resides within lipid rafts. Compartmentalization of Nox1 within lipid rafts in neoplastic cancer cells may represent a unique mechanism for hyperproliferative growth. This hypothesis has been tested using our model EPI and FIB cell lines (Chamulitrat, unpublished data).

## Signal Transduction in Human Cancer Induced by Nox1

Before the discovery of Nox1, ROS generation from NADPH oxidase had already been described in nontransformed and transformed human keratinocyte cell lines[40,41]. Signal transduction in transformed mouse and human keratinocytes involves an activation of activator protein 1 (AP-1) and nuclear factor kappaB (NF-κB) transcription factors that mediate tumor promotion and carcinogenesis[42]. Down-regulation of AP-1 and NF-κB[43] or the removal ROS by catalase[44] suppresses development of tumorigenic phenotypes. Since the discovery of Nox1, a number of studies have supported the role of Nox1 as a mitogenic oxidase in mediating cell proliferation and migration of preneoplastic and cancer cells. Hydrogen peroxide generated by Nox1 mediates cell growth and transformation of NIH3T3 fibroblasts[17], and induces angiogenic switch of human prostate cancer cells[18]. Nox1 targets cyclin D1, which regulates the cell cycle[45]. Accordingly, overexpression of Nox1 increases proliferation concomitant with increased cyclin D1 and extracellular signal regulated kinase (ERK1/2) activity[46].

In mouse fibroblasts, the activated Ras oncogene has been shown to up-regulate Nox1 constitutively by a mechanism of GATA-6 phosphorylation by MEK-ERK, and these processes are necessary for its oncogenic properties[47]. Nox1-generated oxidants in Ras-transformed cells mediate down-regulation of the Rho activity, resulting in disruption of stress fibers and focal adhesions[48]. The down-regulation of Rho activity involves oxidative inactivation of the low-molecular-weight protein-tyrosine phosphatase and a subsequent elevation in the tyrosine-phosphorylated active form of p190RhoGAP, the direct target of the phosphatase[48]. Data in this study are consistent with those showing a correlation between activating Ras mutation and Nox1 overexpression in human colon cancers[12]. Nox1 activity is required for cell growth and survival of a subset of cancer cells, at least in part by activation of proliferative signals, e.g., MAP kinase[47] or c-Src[49]. Upstream of Nox1, ROS production in Caco-2 cells has been shown to be due to the sequential EGF-induced activation of PI3K/β Pix and then Rac1, which then binds to Nox1 to stimulate its oxidase activity[38].

Nox1 has been recently implicated in cell migration of colon adenocarcinoma cancer cells[50]. Nox1 activation by arachidonic acid occurs through 12-lipoxygenase and protein kinase C delta, and controls cell migration by affecting integrin α-2 subunit turnover[50]. Physiologically, Nox1 has been shown to control the directional migration of normal colon epithelial cells[51]. In nonepithelial vascular smooth muscle cells, Nox1 plays a role in cytoskeletal remodeling upon PDGF or FGF treatment, and downstream events involve phosphorylation of cytoskeletal proteins, such as actin, cofilin, and paxillin[52,53]. A recent study has shown that Nox1-dependent ROS contribute to cell invasion by regulating matrix metalloprotease-9 (MMP-9) and cell migration[54]. Nox1 siRNAs inhibit the activation of IKKα kinase, thus suppressing the NF-κB–dependent MMP-9 promoter activity as well as epidermal growth factor–stimulated migration[54].

## CONSTITUTIVE EXPRESSION OF NOX1 IN IMMORTALIZED HUMAN KERATINOCYTES

The role of Nox1 in human cancer has usually been studied in fully transformed cells such as colonic and prostate cancer cells. Research in our laboratory has addressed the role of Nox1 at early steps of transformation of human keratinocytes at the step beyond immortalization. In our initial studies, we observed the presence of Nox1 in spontaneously immortalized HaCaT and human gingival mucosal HPV 16–immortalized keratinocytes (GM16)[55]. Both HaCaT and GM16 cells expressed the phagocyte oxidase cytosolic proteins Rac1, p40*phox*, and p67*phox*. The catalytic flavoheme protein subunit of HaCaT membranes was similar to that of neutrophils. HaCaT membranes, which expressed p22*phox*, showed an absorbance peak at 558 nm indicative of a b-type cytochrome. For kinetic studies, $V_{max}$ values of NADPH oxidase activity of HaCaT membranes were 20-fold lower than those reported for phagocytic oxidase. $K_m$ for NADPH of keratinocyte membranes was higher than that of neutrophil membranes by a factor of two. These kinetic values bolster the notion that keratinocyte NADPH oxidase generates low levels of superoxide, but in a constitutive manner. At mRNA levels, both GM16 and HaCaT cells expressed Nox1, Nox2, and Nox4, while HaCaT cells expressed very low levels of Nox1 mRNA. At protein levels, Nox1 was readily detected in HaCaT, but was nearly undetectable in GM16 cells. This indicates differential rates of Nox1 RNA translation in these two cell lines. Translation to Nox1 protein appears to be more efficient in HaCaT cells than GM16 cells. HaCaT cells are normally cultured in DMEM containing 1.5 mM $Ca^{+2}$, and GM16 cells are cultured in keratinocyte growth medium (KGM) containing 0.05 mM $Ca^{+2}$. It is speculated that Nox1 protein translation may be accelerated by calcium because Nox1 activation is shown to be mediated by intracellular calcium[56]. From these observations, we concluded that Nox1 may a play a role in inducing proliferative advantage under high calcium, which is a differentiation condition of human keratinocytes.

HaCaT cells carry genetic abnormalities from spontaneous immortalization and were identified as a pretransformed phenotype. Significant Nox1 protein expression was found, in non- or pretransformed HaCaT cells, in about the same extent as cancer Caco-2 or HT-29 cells (all proliferate in high-calcium medium). This suggests that Nox1 may render resistance against calcium-dependent differentiation-induced cell death during a pretransformation step. This property is a known criterion for selection of more progressive neoplastic phenotypes[57]. It is known that rodent cells, such as NIH3T3 fibroblasts[58] or rat liver epithelial cells[59], can be easily transformed even upon subculturing at high density. Transformation of human epithelial cells is rare, but requires multiple steps[60]. We therefore adopt this technique in selecting neoplastic cells by culturing them in DMEM containing high calcium, and this is one of the steps in the multistep transformation of human epithelial cells. Calcium in the medium can increase telomerase activity; hence producing immortalized cell lines capable of growing in DMEM[61]. Such selection has been described previously during inhibition of terminal differentiation upon transfection of oncogene Ras[62], HPV 16 E6[63], or treatment with phorbol esters[64].

## CHRONIC ETHANOL EXPOSURE OF IMMORTALIZED HUMAN ORAL KERATINOCYTES GENERATED PRENEOPLASTIC CELLS WITH EMT AND INCREASED NOX1

By using the multistep carcinogenesis concept, we first tested whether Nox1 increases neoplastic progression of immortalized human epithelial cells and, if so, whether this process is reversible and/or generates a more invasive phenotype. By selection of cells under a growth constraint, i.e., calcium-induced differentiation, we expected that resistant or selected cells would exhibit increased rates of translation of the Nox1 protein. This will allow them to proliferate in high-calcium medium. This study design will shed light on a possible role of Nox1 in an early transformation step beyond the immortalization step.

Human gingival mucosal keratinocytes immortalized with HPV 16 E6/E7 oncogenes (GM16) were utilized as the starting point of the transformation scheme[65]. In our first study, chronic ethanol treatment was chosen for its applicability to ethanol-induced oral cancer (Fig. 2). GM16 cells were cultured in low-calcium keratinocyte medium and exposed to 30 mM ethanol in a closed incubator with ethanol replenishment every week for 9 weeks. The medium of ethanol-treated cells was then changed to DMEM, which caused differentiation-induced cell death, leaving selectable resistant cells to survive and proliferate. Without ethanol treatment or with 3- or 6-week ethanol exposure, no surviving GM16 cells were obtained in DMEM, indicating that prolonged ethanol exposure as a growth constraint was indeed required. Therefore, prolonged stationary cultures can be used to select preneoplastic human cells, which will allow genetic changes in cells affected by ethanol. Prolonged subcultures (~6–8 months) were carried out in DMEM to obtain sufficient proliferative cell populations, which were separated into two cell phenotypes (Fig. 2). One with epithelium-like morphology (EPI cells) grew anchorage dependently, and the other with spindle-shape fibroblast-like morphology (FIB cells), indicating that these cells had undergone EMT[65]. FIB cells, but not EPI cells, exhibited anchorage-independent growth (AIG). In comparison to EPI cells, the EMT characteristics of FIB cells were characterized by a decrease in the expression of keratins, desmoplakins, and a complete loss of K14. EMT of FIB cells also correlates with an increase in the expression of vimentin and simple epithelial keratin K18. A recent study demonstrated that ROS are a crucial factor to induce the cell-cell dissociation, an initial step of EMT, but does not provide sufficient signals to establish and maintain the EMT[66]. Chronic ethanol treatment combined with the prolonged subcultures may contribute to EMT stability of FIB cells. FIB cells at low passages ($p < 22$) were unstable, being able to morphologically and functionally revert back to EPI phenotype upon adapting cultures in low-calcium medium, while no reversion was observed in FIB cells at high passages ($p > 43$)[67]. Thus, FIB cells at $p < 22$ may be used to test the ability of anticancer drugs to reverse to a nontransformed phenotype.

Both EPI and FIB cells (at their 60–70 passages) did not form tumors in nude mice; thus, they were not yet tumorigenic (FIB cells produced benign cysts). Both EPI and FIB cells were of benign phenotype. FIB cells were more transformed than EPI cells, while EPI cells were more transformed than GM16 cells. Upon passaging, FIB cells acquired the ability to grow in low or an absence of serum, showing a proliferative capacity more than EPI cells (Fig. 3A). EPI and FIB cells were therefore used as model cells to study early steps of transformation in association with Nox1 expression.

## Increased Nox1 Expression in FIB Cells

EPI and FIB cells were used as model cells (FIB being more transformed with AIG) to find an association with Nox1[36]. Nox1 protein levels were determined in membrane fractions. Compared with EPI membranes, FIB membranes possessed a 63-kDa Nox1 protein at higher levels and exhibited a 2.8-fold higher capability for superoxide and hydroxyl radical generation. Both EPI and FIB cells expressed more abundant Nox1 protein at a proliferating stage than that at a quiescent confluent phase. Nuclei isolated from EPI and FIB cells contained a 63-kDa Nox1 protein. Compared with EPI cells, FIB cells expressed elevated levels of Jun N-terminal kinase (JNK) and ERK proteins. JNK2 was constitutively phosphorylated in FIB cells. Nox1 expression in FIB cells appeared to increase upon passaging up to $p > 150$. Thus, an association of Nox1 was found in preneoplastic keratinocytes that exhibited EMT. Furthermore, Nox1-dependent ROS mediate in redox-sensitive ERK and JNK signaling at an early stage of transformation beyond the immortalization step of human keratinocytes.

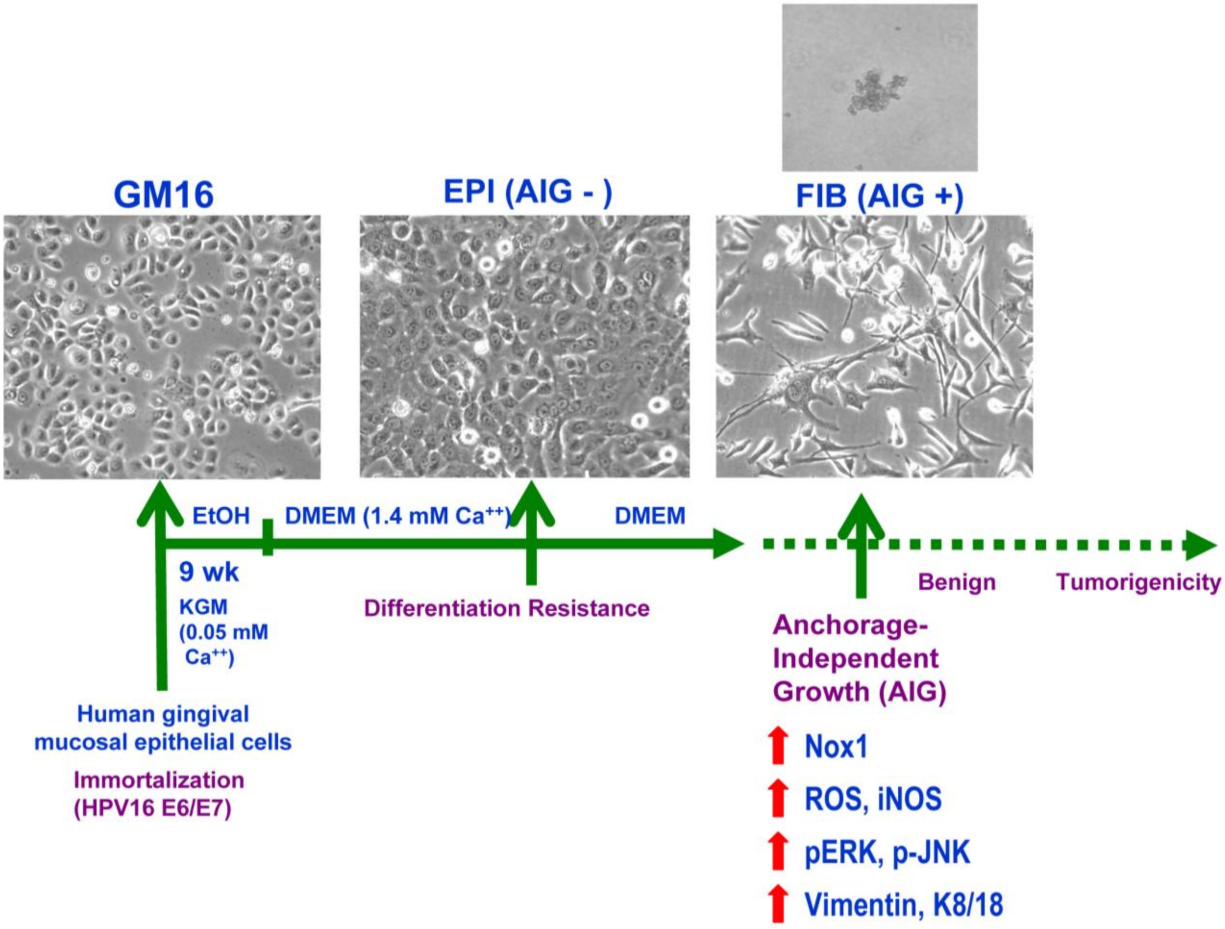

**FIGURE 2.** An experimental design to determine an association of Nox1 on cell transformation induced by ethanol stress. Immortalized human gingival mucosal keratinocytes (GM16) were exposed to ethanol for 9 weeks, followed by exposure in high calcium in DMEM. Differentiation-resistant cells were resultant preneoplastic cells; EPI and FIB cell lines. FIB cells were capable of growing anchorage independently, thus being more transformed than EPI cells. FIB cells expressed increased Nox1, ROS, and iNOS (inducible nitric oxide synthase) concomitant with an activation of ERK and JNK. Concerning cytoskeletal changes, FIB cells expressed vimentin and simple/fetal epithelial keratins K8/18.

## Increased iNOS Expression in FIB Cells

Hydrogen peroxide is known to induce inducible nitric oxide synthase (iNOS), which is a downstream target gene associated with cancer progression[68]. iNOS is shown to be associated with tumor invasiveness[69]. We studied the expression of redox-sensitive iNOS in these EPI and FIB cells[67]. iNOS at mRNA and protein levels was up-regulated in FIB cells in comparison with EPI cells. FIB cells at low passages ($p < 22$) were unstable, being able to revert back to the EPI phenotype morphologically and functionally, while no reversion was observed in FIB cells at high passages ($p > 43$). The morphological reversion of FIB cells was associated with the reversal of vimentin expression as well as AIG. More importantly, these revertants showed reduced levels of iNOS mRNA as well as MAP kinase ERK and phospho-ERK protein expression, while FIB cells without reversion maintained the expression. The MEK1/2 inhibitor U0126 could reduce detectable iNOS mRNA levels, suggesting that MAP kinases were upstream regulators of iNOS transcription. U0126 caused both morphological and functional reversion of FIB cells, indicating involvement of MAP kinases in these functions.

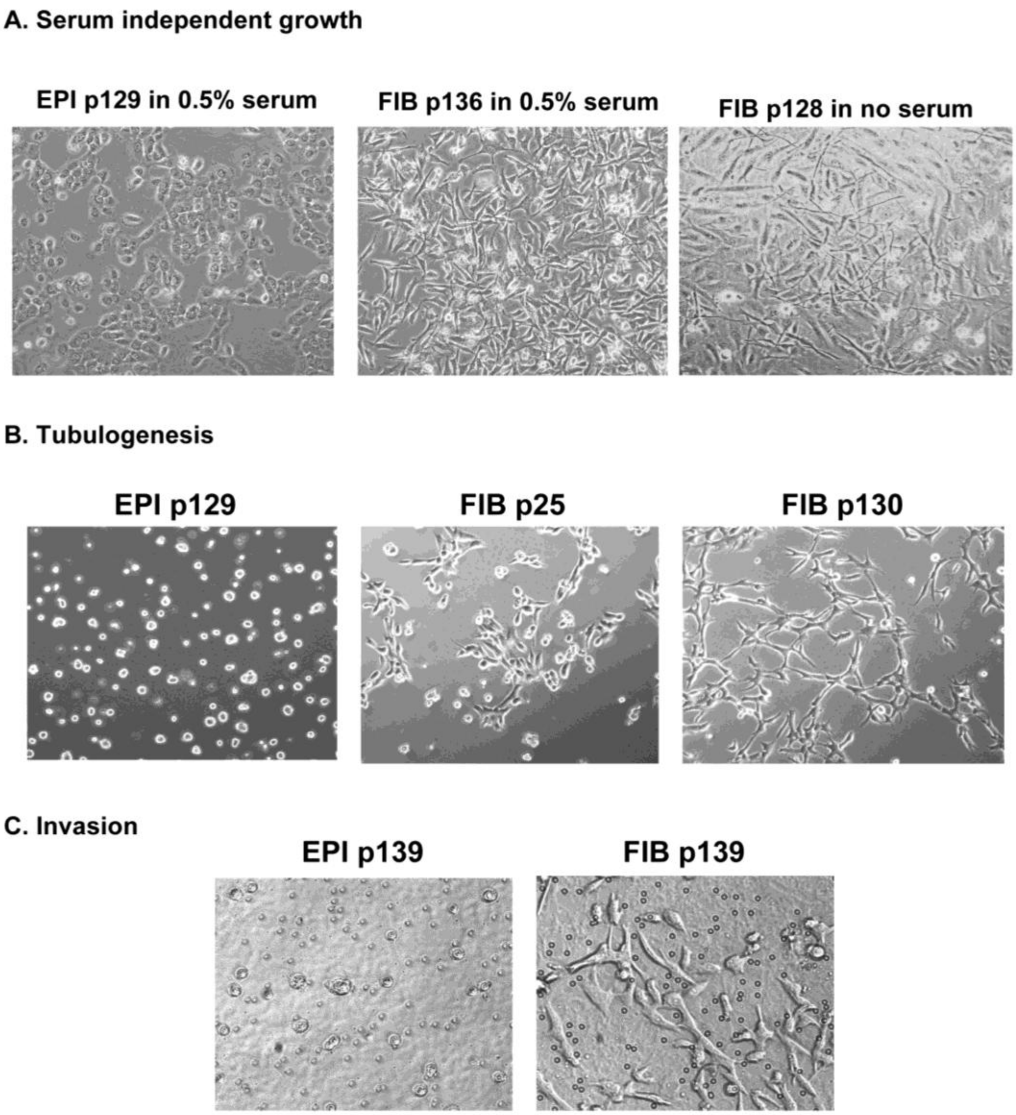

**FIGURE 3.** Transformation characteristics of FIB cells compared with EPI cells. (A) FIB cells exhibited serum-independent growth better than EPI cells. FIB cells were more proliferative in low and no serum cultures. FIB cells became even more spindle shaped when cultured in no serum. (B) Formation of tubulogenic morphogenesis of FIB cells on Matrigel. EPI and FIB cells at p 129–130 were plated on prepared Matrigel-coated dishes for 4 days in DMEM containing 1% FCS. FIB cells formed tubules (cords without lumen) with extensive meshwork. (C) By using Boyden chamber assay, FIB cells invaded through filters better than EPI cells.

## Branching Morphogenesis of FIB Cells

Nox1 has been shown to be associated with branching tubulogenic potential as demonstrated in sinusoidal endothelial cells[70]. Indeed, branching morphogenesis is used to characterize breast[71] and prostate[72] cancer cells. We therefore used branching morphogenesis as an indicator of neoplastic transformation. Upon resistance against Matrigel-induced differentiation, FIB cells formed branching tubulogenesis with cords lacking lumen (Fig. 3B). EPI cells formed unproliferative rounded cells as they died off from differentiation when plated on Matrigel. The ability of FIB cells to form branching tubulogenesis bolsters its more transformed phenotype with EMT characteristics.

## Invasiveness and AIG in FIB cells

ROS have been shown to increase invasive potentials of transformed cells[73]. Nox1-mediated ROS have been recently shown to regulate invasiveness of Ras-induced mouse transformed fibroblasts[54]. FIB cells were AIG positive, being able to grow on semi-solid agar at 2% efficiency, while EPI cells required anchorage for growth[65]. The EMT phenotype of FIB cells suggests that they are more invasive and metastatic than EPI cells. Using the Boyden invasion chamber, we showed that FIB cells had significant migration through the filters, while EPI cells did not migrate (Fig. 3C). For application purposes, FIB cells were used as model cells for the testing of 2,3-dehydrosilybin (DHS) as an anticancer agent for an inhibition of invasion[74].

## Enhanced Migration and MMP-2 and MMP-9 Releases in FIB Cells

ROS are known to regulate proteins that are important for degradation of extracellular matrices, such as gelatinase matrix MMP-2 and-9, which are key enzymes for degrading type IV collagen and thought to play a critical role in tumor invasion and metastasis[75]. EPI cells secreted latent MMP-9 (92 kDa) (Fig. 4A). FIB cells released a protein at 86 kDa that was not the active MMP-9 (82 kDa). MMP-9 at 86 kDa is known as an intermediate after first cleavage of latent MMP-9 by MMP-3[76]. FIB cells thus secrete partially active MMP-9 by the action of stromelysin MMP-3, and the implication of MMP-3 in FIB cells is consistent with recent data showing the role of ROS-induced MMP-3 in EMT induction and genomic instability[77]. In the study we carried out to test anticancer effects of DHS[74], EPI and FIB cells were used to determine inhibitory effects of gelatinase (MMP-2 and MMP-9) activities. Expression of MMP-9 has been associated with cell migration of Ras-transfected keratinocytes[78]. We therefore performed wound assays of EPI and FIB cells. FIB cells were capable of migrating into wounds better than EPI cells (Fig. 4B). This bolsters FIB cells as a more transformed phenotype exhibiting greater expression of partially active MMP-9 and cell migration.

## Sensitivity of EPI and FIB Cells upon Treatment with Anticancer Drugs

EPI and FIB cells arose from the same “mother” GM16 cells, with FIB cells being more transformed than EPI cells. Therefore, these cell lines are valuable for testing the effectiveness of anticancer agents. We carried out a comparison study for effectiveness of silybin and DHS by using EPI and FIB cells either by direct treatment[74,79] or sensitization with TNF-$\alpha$[79]. We found that test agent DHS induced apoptosis in FIB cells, but not in EPI cells. Apoptosis induced by DHS in FIB cells was associated with loss of mitochondrial membrane potentials. Thus, in terms of apoptosis induction by a test agent, EPI and FIB cells may be used to compare anticancer efficacy and specificity of drugs towards a more transformed phenotype.

## Association of Antiapoptosis Protein Expression with EMT of FIB Cells

It has been recognized that inhibitors of apoptosis proteins (IAPs), such as survivin, are markers of multistep malignant development[80,81]. We analyzed expression of survivin and IAPs in EPI and FIB cells (Fig. 5). We found differential expression showing FIB cells expressing increased survivin and c-IAP-1 more than EPI cells. No difference in c-IAP-2 was detected. This appears to correlate with higher proliferative growth and EMT, and invasive phenotype of FIB cells. The latter was studied in detail using two more EMT cell lines[82].

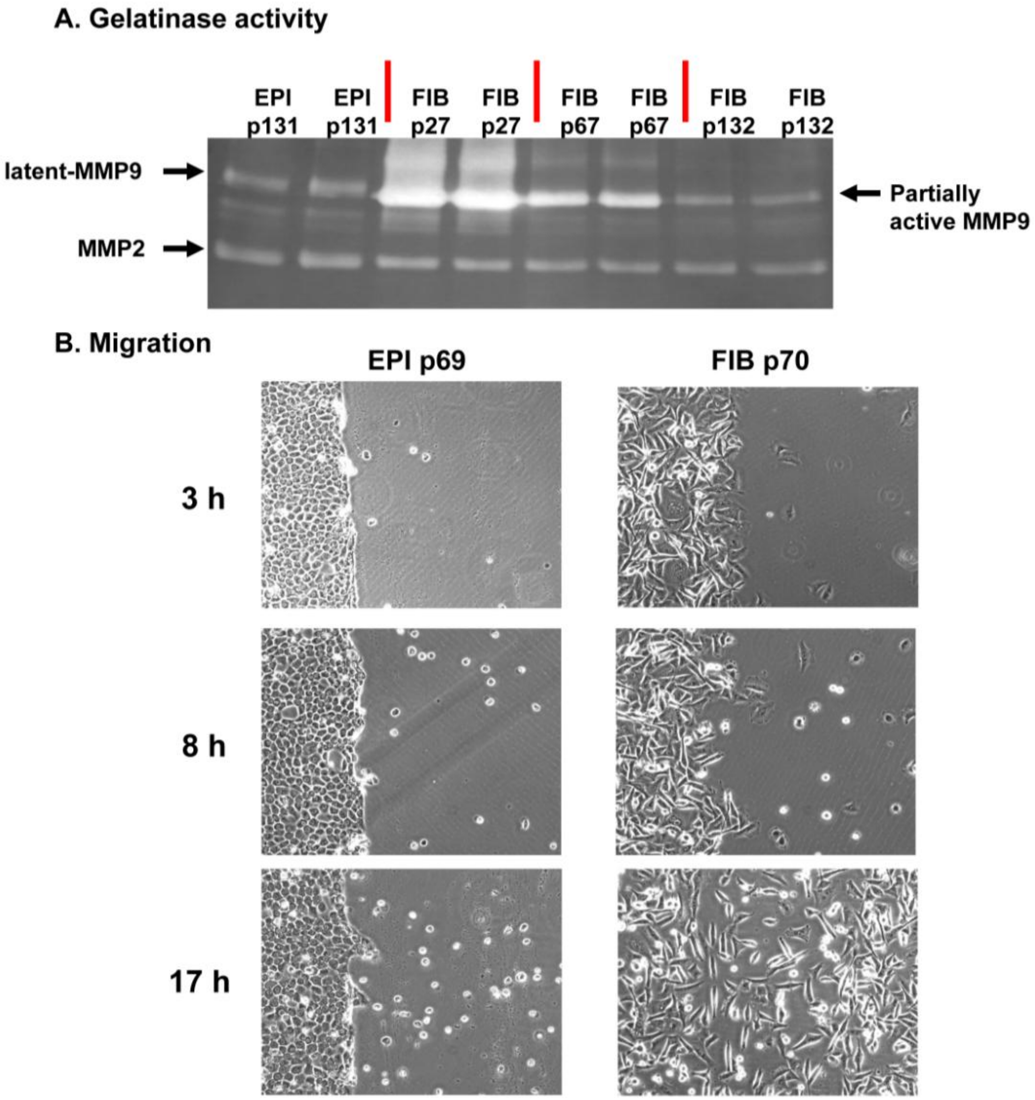

**FIGURE 4.** Cellular motility of EPI and FIB cells. (A) Gelatinase activity of EPI and FIB cells in conditioned medium after culture for 24 h. FIB cells released partially active MMP-9, while EPI cells released latent MMP-9. (B) In wound assay, FIB cells were more migratory than EPI cells.

## Organotypic Cultures of EPI and FIB Cells

EPI and FIB cells were analyzed in a three-dimensional tissue/epithelial context with respect to the stratified gingival epithelium[83]. In epithelial equivalents, FIB cells exhibited a significant down-regulation in K14 and integrin α6 protein, and a loss of E-cadherin, whereas vimentin was increased. FIB epithelial equivalents were devoid of transcripts for E-cadherin. The FIB phenotype exhibited a poor epithelial structure, coinciding with disturbances in the expression of epithelial biomarkers and the persistence of mesenchymal vimentin. Our data indicate that FIB cells share features of EMT and reflect a more progressive stage in epithelial cell transformation.

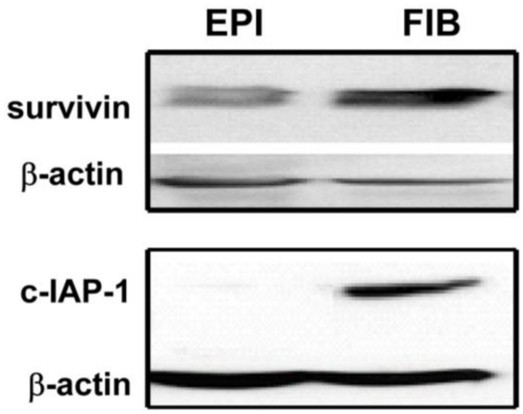

**FIGURE 5.** Survival and antiapoptosis expression in EPI and FIB cells. FIB cells expressed increased survivin and c-IAP-1 proteins at higher levels than EPI cells.

## NOX1 TRANSFECTION OF IMMORTALIZED HUMAN KERATINOCYTES GENERATED PRENEOPLASTIC PROGENITOR CELLS

We demonstrated that chronic ethanol treatment for 9 weeks rendered selectable preneoplastic EPI and FIB cells, with FIB cells exhibiting EMT and expressing increased Nox1. Thus, an association of Nox1 with preneoplastic EMT cells has been demonstrated. To find whether the EMT phenotype is the result of Nox1 overexpression was our next step[84]. We performed cloning of pcDNA3.1 containing Nox1. Transfections of GM16 keratinocytes with Nox1 plasmid or plasmid alone by using nucleofection or Fugene 6 transfection were carried out**.** To keep conditions similar to chronic ethanol treatment, we kept transfected cells in culture for a total of 8 weeks with trypsinization only once after the first 4 weeks. Transfected cells were then split 1:2 with one flask for continuation in KGM culture and the other for selection by culturing in DMEM.

Within 7–10 days after DMEM exposure, Nox1-transfected cells produced fast dividing resistant cells. After three more confluence rounds, transfected cells selected in DMEM persistently gave rise to proliferative progenitor cells, so-called Nox1 lines. Five Nox1 lines were reproducibly generated from Nox1-transfected cells: the NuB1 line (first line resulting from nucelofection transfection) was the only line with EMT, and other lines were non-EMT cells. Plasmid transfections did not produce any selectable cells in DMEM. Nox1 lines showed differential mRNA expression of Nox1, Rac1, p47*phox*, p67*phox*, NoxO1, and NoxA1 with concomitant increased superoxide generation upon EGF treatment. All five Nox1 lines contained varying amounts of E-cadherin, involucrin, vimentin, and keratins K8/K18. Four non-EMT lines and EPI cells expressed K18, while the EMT NuB1 (similar to FIB) cells expressed vimentin and K8/K18, indicating that this line may be in preneoplastic progression. Our data demonstrated that Nox1 accelerated neoplastic-like progression by inducing generation of progenitor cells. NuB1 indeed expressed antiapoptotic proteins cIAP-1, Bclx, and cdkinase inhibitor $p16^{INK4a}$ and $p27^{kip1}$ at higher levels than the four non-EMT lines[82]. Thus, NuB1 was the line with an enhanced neoplastic progression similar to FIB cells that were obtained from chronic ethanol treatment of GM16 cells.

## CONCLUSIONS

Our results from chronic ethanol treatment[36,65] and Nox1 overexpression[84] have emphasized the importance of Nox1 in inducing resistance against differentiation-induced cell death and generating selectable preneoplastic human keratinocytes. Our data have also indicated that Nox1 and its oxidants contribute to the progression of human keratinocytes during the early stage of cell transformation. As demonstrated in human oral mucosal keratinocytes, Nox1 may also exhibit a capacity to induce an EMT phenotype demonstrating AIG and invasiveness. Finally, the progenitor Nox1-containing cell lines represent model cell lines for comparative investigation between EMT and non-EMT phenotypes, as well as for cytotoxicity tests of anticancer agents.

## PERSPECTIVES

In both studies from chronic ethanol treatment and Nox1 overexpression, progenitor cells with epithelial morphology (non-EMT cells) showed persistent expression of K18, whereas the EMT expressed both K8 and K18. Further questions arise whether Nox1 would play a role in the regulation of these epithelial keratins and, if so, whether the regulation is at transcriptional or post-translational levels. While current studies have demonstrated that Nox1 is able to increase proliferative potentials (i.e., overcoming senescence) of immortalized cells, these Nox1-driven progenitor cells were in the step of enhanced preneoplastic progression. In our experiments, the generation of progenitor cells from immortalized cells arises not just from the presence of Nox1, but also the requirement of prolonged stationary cultures. This implies that transfection of primary human keratinocytes with Nox1 may extend lifespan, but may not be sufficient to elicit immortality (unless other factors such as Ras are introduced). Thus, Nox1 by itself cannot be considered as an oncogene of human epithelial cancer, but rather a significant contributor or a cofactor of early transformation progression steps in the multistep carcinogenesis.

## ACKNOWLEDGMENTS

The author's research work was supported by the Deutsche Forschungsgemeinschaft (CH288 3-3), Thailand Research Fund/DAAD, and Paul-Erhlich-Society for Chemotherapy. The help of students and trainees W. Chunglok, A. Huber, L. Ersoy, P. Thongphasuk, and A. Sattayakhom is acknowledged.
